# Testing eye temperature assessed with infrared thermography to evaluate stress in meat goats raised in a semi-intensive farming system: a pilot study

**DOI:** 10.5194/aab-62-199-2019

**Published:** 2019-04-16

**Authors:** Ester Bartolomé, Florencia Azcona, María Cañete-Aranda, Davinia I. Perdomo-González, Joana Ribes-Pons, Ester M. Terán

**Affiliations:** 1 Dpto. Ciencias Agroforestales, ETSIA, Universidad de Sevilla, Carretera de Utrera, Km. 1. C.P. 41013 Sevilla, Spain; 2 Universidad de Córdoba, Campus Universitario de Rabanales, Carretera Nacional IV, Km. 396. C.P. 14014 Córdoba, Spain

## Abstract

The Blanca Serrana goat is selected for meat production and usually raised in an extensive farm system. The meat goat industry is getting bigger in Spain, evolving to more intensive farming systems. The negative influence of stress produced by daily management on animal welfare is even bigger in these animals as they are not used to getting so close to humans. Eye temperature has recently appeared as an appropriate and noninvasive tool for welfare assessment in cattle, but no previous studies have been developed in goats. Thus, the main aim of this pilot study was to test eye temperature as a noninvasive tool to explore stress levels associated with a semi-intensive farming system for meat goats in comparison with the standard measurements of stress.

For that, 24 Blanca Serrana goats were used. Heart rate (HR), respiratory rate (RR) and eye temperature (ET), assessed with infrared thermography samples, were collected just before and just after a stressful situation created to check how the routine management of semi-intensive farming systems affected this species. A factorial ANOVA, least square means and Scheffé post hoc comparison analyses found statistically significant differences due to the stress test moment for RR (p<0.05) and ET (p<0.001) with higher values shown after the stress test than before it. Differences due to age were found just for HR (p<0.05) and RR (p<0.01) stress parameters, with kids showing higher results than adults. Pearson correlations between HR, RR and ET parameters showed a medium–high positive correlation of 0.56 between RR and ET.

Thus, ET appears as an appropriate and noninvasive tool to explore stress levels associated with a semi-intensive farming system for meat goats.

## 1 Introduction

Stress in farm animals is a reaction that occurs when animals are exposed to adverse environmental conditions, producing many unfavorable consequences, from discomfort to death (Etim et al., 2013). The influence of stress is widely studied in dairy goats, being one of the factors with greater influence on animal welfare, milk yield and health status (Salak-Johnson and McGlone, 2007; Hamzaoui et al., 2013). On the other hand, milk yield is positively correlated with a calm temperament. Thus, as dairy goats have been selected for milk yield, they have also been selected indirectly to show lower levels of stress during lactation (Salama et al., 2014). These animals are mainly bred in an intensive farming system, with goats living in pens with limited or no access to bare yards and being milked twice a day. Furthermore, they are used to daily physical contact with humans and management due to lactation. On the other hand, the number of studies developed to assess welfare and stress levels in meat goats is scarce, probably due to the extensive farming system associated, with little human contact and almost no mechanization (González-Martínez et al., 2014).

The Blanca Serrana goat is classified as a Spanish native breed, with a total number of 4288 females and 454 breeding males in 2017 (MAPAMA, 2016). Generally, these goats selected for meat production and usually raised in an extensive farm system, with very little management from the farmer as they spend most of their time grazing free on the mountains (Camacho et al., 2005). Recently, the meat goat industry has been getting bigger, with Spain having the second largest (14.2 %) in the entire European Union territory after Greece (24.8 %) (FAOSTAT, 2018). The sector will soon demand bigger and more specialized farms that can handle the consumer demand associated with a semi-intensive farming system, which is still linked to the land but more mechanized. Hence, regarding the Blanca Serrana goat breed, raising these animals on a more intensive farm system would definitely affect their welfare as no previous selection has been made for stress or fear attenuation in this breed.

Different methodologies have been developed for stress assessment in animals, such as cortisol levels, heart rate, respiratory rate or catecholamine level (Moberg, 2000). However, the techniques used to obtain them (blood draw, touching the animal, etc.) can be stressful themselves, hence, biasing the stress measures obtained (Stewart et al., 2005) and even more so in animals that are not used to human management. Hence, the use of minimum invasive methodologies for stress assessment would be advisable to assess animal welfare in this species. For that, eye temperature (ET) assessed with infrared thermography has lately appeared as an appropriate tool for stress and welfare assessment in other ruminants (Stewart et al., 2007), but no studies have been developed in goats yet.

Thus, the main aim of this pilot study was to test eye temperature as a noninvasive tool to explore stress levels associated with a semi-intensive farming system for meat goats in comparison with the standard measures of respiratory and heart rates.

## 2 Material and methods

### 2.1 Animals and location conditions

For this study, 24 Blanca Serrana goats (42 % goat kids and 58 % adults) were used, with ages ranging from 25 to 30 d old for goat kids and 4 to 6 years old for adult females.

Samples were taken on the experimental farm facilities of the Veterinary Faculty of the University of Cordoba (Spain). The study was carried out in April 2018 on the same day, with sunny weather and temperatures oscillating between 23 and 27 ${}^{\circ }$C and relative humidity from 33 % to 42 %. Animals were housed in a covered 20×20 m pen, with a 10×10 m bare yard. It also included a 1×1 m crowd pen which the animals entered for routine management practices (veterinary treatments, physiological test, etc.).

The experiment was carried out in the same pen where the animals were usually located, assuring a calm and familiar environment before undergoing the stress test. They were fed with hay, concentrate and water ad libitum, as usual.

### 2.2 Physiological data

The stress response of the animals was measured with ET assessed with infrared thermography as a novel and noninvasive tool. Heart rate (HR) and respiratory rate (RR) measurements were taken as a reference of regular stress measurements. Samples were collected just before (at rest) and just after a stressful situation created for this study, in order to check how the routine management of semi-intensive farming systems affected this breed. The stressful situation (or stress test) was created according to routine management practices that are held in semi-intensive and intensive farm systems (González-Martínez et al., 2014). It consisted on a person walking around and within the animals for 1 min, making noise and waving his arms as he guided the animals to another pen located nearby. All physiological measures were recorded individually, immediately before and after animals entered the crowd pen, where they were located after the stress test, so that only minimal handling of the animals was necessary. It has to be noticed that, despite it was the least invasive procedure to approach the animals, this procedure itself produces stress, thus biasing the obtained results.

**Figure 1:**
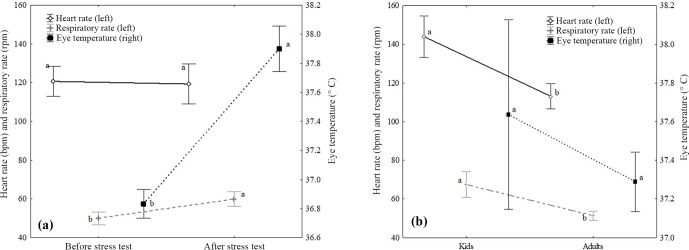
Least square means analysis (mean ± standard error) and Scheffé post hoc comparison test (indicated with letters) for the stress variables with statistically significant results in the ANOVA test due to the stress test moment **(a)** and the age of the animals **(b)**. Different letters indicate statistically significant differences (p<0.05) between means.

Eye temperature images were taken with a portable infrared thermography (IRT) camera (ThermaCam i70 0, FLIR Systems AB, Danderyd, Sweden). In order to calibrate the camera results, environmental temperature and relative humidity were recorded with a digital thermo-hygrometer (Extech^®^ 44550) every time an eye temperature sample was taken. To determine eye temperature, an image analysis software Therma Cam Researcher Pro 2.8 SR-2 (FLIR Systems AB, Sweden) was used, measuring the maximum temperature (${}^{\circ }$C) within an oval area traced around the medial posterior palpebral border of the lower eyelid and the lacrimal caruncle. This maximum eye temperature was used for the analyses. The left eye of all the goats was scanned from a 90${}^{\circ }$ angle and at a distance of 1 m. Two images were taken per animal. Later, the mean value of both photos was used for the analyses.

RR was assessed visually during 15 s, observing the movement of the animal's rib cage, and then measures were multiplied by 4 to be quantified as breaths per minute (bpm).

HR was assessed during 15 s with a standard stethoscope (Littmann Classic 3M^®^), and then measures were multiplied by 4 to be quantified as heart beats per minute (ppm).

ET images, RR and HR were collected twice, before (BST) and after (AST) the stress test. Furthermore, in order to avoid cross-stress effects due to the methodology used to obtain the samples, these were taken from least to most invasive: first ET, then RR and finally HR.

All the procedures used in this study complied with the animal ethical guidelines published by the International Society for Applied Ethology and met the International Guiding Principles for Biomedical Research Involving Animals.

### 2.3 Statistical analyses

Shapiro–Wilk tests of the collected data revealed no deviation from normality for all the stress variables used (results not shown), so parametric statistics were used.

The possible influence of the effects “age” (goat kid, adult female), “stress test moment” (BST or AST) and “age–stress × test moment interaction” (goat kid–BST, adult–BST, goat kid–AST, adult–AST) on the physiological measurements (ET, HR, RR) was assessed with a factorial ANOVA procedure. Then, a least-squares means (LSM) analysis and a Scheffé post hoc comparison test were performed for statistically significant effects (p<0.05). Finally, in order to check the relation between the stress variables used in this study, a Pearson's correlation test was performed.

Statistical analyses were performed using the Statistica v. 12.0 package (StatSoft, 2012).

## 3 Results

The descriptive analysis (Table 1) showed mean HR values of 120 ppm, mean RR values of 55 bpm and mean ET values of 37.4 ${}^{\circ }$C for the meat goats analyzed in this study. Coefficients of variation were the highest for HR, with 22 % of variation, followed by RR with 21 % and by ET with less than 2 % of variation.

**Table 1:** Descriptive statistics for heart rate (HR), respiratory rate (RR) and eye temperature (ET) measurements obtained for the meat goats analyzed.

	Mean ± SE	Min	Max	C.V. (%)
HR	120.00±6.29	60.00	162.00	22.23
RR	55.00±2.75	42.00	78.00	21.25
ET	37.37±0.16	36.30	38.85	1.78

SE: standard error; min: minimum; max: maximum; C.V. (%): coefficient of variation.

**Table 2:** Factorial ANOVA for every stress variable considered, due to age and stress test moment factors, including the age×stress test moment interaction factor.

Variables/	Intercept		Age		Stress test moment		Age×stress test moment	
factors	F	significance	F	significance	F	significance	F	significance
		level		level		level		level
HR	323.70	${}^{***}$	4.66	${}^{*}$	0.06	n.s.	0.36	n.s.
RR	594.48	${}^{***}$	10.86	${}^{**}$	5.84	${}^{*}$	0.43	n.s.
ET	136 400.30	${}^{***}$	2.90	n.s.	36.80	${}^{***}$	2.10	n.s.

HR: heart rate; RR: respiratory rate; ET: eye temperature; n.s.: not statistically significant; ${}^{*}$ p<0.05; ${}^{**}$ p<0.01; ${}^{***}$ p<0.001.

The ANOVA analysis (Table 2) showed statistically significant differences in HR just for the age effect (p<0.05); in ET for the stress test moment effect (p<0.001) and in RR for both effects (p<0.01 and p<0.05, respectively). None of the variables analyzed showed statistically significant differences for the age–stress-test-moment interaction.

**Table 3:** Pearson correlations between the stress parameters: heart rate, respiratory rate and eye temperature.

	Respiratory rate		Eye temperature	
	Correlation	Significance	Correlation	Significance
		level		level
Heart rate	0.39	n.s.	0.14	n.s.
Respiratory rate	–	–	0.56	${}^{*}$

n.s.: not statistically significant; ${}^{*}$ p<0.05; ${}^{**}$ p<0.01; ${}^{***}$ p<0.001.

Mean differences were analyzed with a LSM and a Scheffé post hoc analyses for both effects that showed statistically significant differences for any of the stress variables analyzed (Fig. 1). Thus, for the stress test moment effect (Fig. 1a), animals showed higher RR and ET values after the stress test than before it, with a mean difference of 10 bpm and 1.1 ${}^{\circ }$C between test moments, respectively. According to the age factor (Fig. 1b), kids showed higher HR and RR values compared to adults, with a mean difference of 26 and 20 bpm for HR and RR, respectively, between ages.

Correlations between stress parameters are shown in Table 3. Only a statistically significant and positive correlation of medium–high magnitude (0.56) was found between RR and ET.

## 4 Discussion

Meat goats are mainly bred in extensive farming systems, where very little management and human contact is needed (González-Martínez et al., 2014). Thus, no selection has been made on these animals to avoid stress due to daily management. The Spanish meat goat breed, Blanca Serrana, is an example of it, showing in general, an extensive management farming system (Camacho et al., 2005). However, the population showed in this study was raised in a semi-intensive farming system, which implies a certain human contact which these animals are not used to. Thus, they could develop a stress response that would later affect their welfare status and, hence, their meat production. Measuring this stress response in these reactive animals is quite complicated due to any classical approach being quite invasive, hence producing stress itself.

For that, ET was used as a noninvasive tool to explore stress levels in these animals in comparison with the standard measures of RR and HR. The latest techniques have been frequently used to assess stress in animals (Moberg, 2000). On the other hand, eye temperature assessed with infrared thermography is a new tool that has been already proven as an appropriate technique for stress assessment in other animal species, such as horses (Valera et al., 2012), cattle (Stewart et al., 2005), dogs (Travain et al., 2015) or even rabbits (De Lima et al., 2013). However, although infrared thermography has been used to assess stress by measuring rectal or average surface temperature in goats (Da Silva et al., 2014), no previous studies have been developed in this species for stress assessment using the caruncle temperature. This anatomical area has rich capillary beds innervated by the sympathetic system and responding to changes in blood flow (Stewart et al., 2007). Hence, during an acute stress response, ET tends to increase, possibly due to an increased dilation of the ocular blood vessels and an increased visual attention or orientation (Yarnell et al., 2013).

HR values (Table 1) showed a wider range than those obtained in previous studies developed in meat goats (Puchala et al., 2009; Ja'afaru et al., 2019).

Furthermore, results in Table 2 showed that this new parameter (ET) was not affected by the age of the animals but by the stress test moment, showing good potential as an appropriate tool to explore the stress levels associated with a semi-intensive farming system for meat goats.

Results in Table 3 showed a statistically significant increase in the ET and RR stress parameters after the stressful stimulus was introduced, but not of the HR. This could be due to a fear and anxiety response associated with the activation of an acute stress response (Fell et al., 1985) which comprises the activation of the hypothalamic–pituitary–adrenal (HPA) axis (Dinan, 1996). These findings support previous studies that related changes in ET to the activation of the HPA axis either in horses (Valera et al, 2012) or in cattle (Stewart et al., 2009).

Considering the age effect, only HR and RR showed statistically significant differences, with goat kids showing higher HR and RR values than adults. This could be due, first, to differences in metabolic rates (higher in kids), which comprises a higher oxygen consumption, hence influencing both heart and respiratory rates (Al-Tamimi, 2007). Secondly, adults are more used to new stimuli, hence developing a smaller physical and physiological activation of the autonomous nervous system (ANS) than goat kids, showing increased HR and RR values (Moberg, 2000).

Finally, the correlation results, together with ANOVA findings, support previous studies developed in horses (Bartolomé et al., 2013) or cattle (Stewart et al., 2009), indicating that RR and ET could be measuring a stress reaction more related to reactivity and temperament, whereas HR could be measuring a stress more related to the physical condition and physiological state of the animal. However, due to the limited nature of the experiment performed, further studies should be developed in meat goats to confirm these relations.

The results obtained in this study showed that meat goats raised in semi-intensive farming systems showed stress levels associated with human management that could be adequately assessed with ET measured using infrared thermography. Hence, this shows that this new tool is a useful, easy and noninvasive method to explore stress levels in meat goats.

However, in order to determine whether this stress produced by management procedures affects an animal's welfare, further studies should be developed comparing these results with meat quality and production parameters.

## Data Availability

For data, contact ebartolome@us.es.
